# Irreversible electroporation versus radiotherapy after induction chemotherapy on survival in patients with locally advanced pancreatic cancer: a propensity score analysis

**DOI:** 10.1186/s12885-019-5607-3

**Published:** 2019-04-27

**Authors:** Chaobin He, Jun Wang, Shuxin Sun, Yu Zhang, Xiaojun Lin, Xiangming Lao, Bokang Cui, Shengping Li

**Affiliations:** 1 Department of Hepatobiliary and Pancreatic Surgery, State Key Laboratory of Oncology in South China, Collaborative Innovation Center for Cancer Medicine, Sun Yat-sen University Cancer Center, Guangzhou, 510060 China; 2 Department of Ultrasonics, State Key Laboratory of Oncology in South China, Collaborative Innovation Center for Cancer Medicine, Sun Yat-sen University Cancer Center, Guangzhou, 510060 China; 3 State Key Laboratory of Ophthalmology, Zhongshan Ophthalmic Center, Sun Yat-sen University, Guangzhou, Guangdong 510060 People’s Republic of China

**Keywords:** Locally advanced pancreatic cancer, Irreversible electroporation, Chemotherapy, Radiotherapy, Efficacy

## Abstract

**Background:**

Locally advanced pancreatic cancer (LAPC) represents more than one third of pancreatic cancers and owns poor survival after the standard chemotherapy. Irreversible electroporation (IRE) is a novel method and has been recently used in LAPC. The aim of this study was to compare the efficacy of IRE and radiotherapy after induction chemotherapy for patients with LAPC.

**Methods:**

From August 2015 to August 2017, a total of 76 patients with biopsy proven LAPC and who had received IRE or radiotherapy after chemotherapy were included. Thirty-two pairs of patients were selected through propensity score matching (PSM) analysis and the efficacy of two treatments was compared.

**Results:**

Before PSM analysis, after induction chemotherapy, patients with LAPC benefited more in terms of overall survival (OS) and progression free survival (PFS) from IRE, compared with radiotherapy (2-year OS rates, 53.5% vs 26.9%, *p* = 0.039; 2-year PFS rates, 28.4% vs 13.3%, *p* = 0.045). After PSM analysis, the survival benefits of OS and PFS of patients after induction chemotherapy followed by IRE were more obvious than those of patients treated with radiotherapy (2-year OS rates, 53.5% vs 20.7%, *p* = 0.011; 2-year PFS rates, 28.4% vs 5.6%, *p* = 0.004). Multivariate Cox regression analysis indicated that IRE after induction chemotherapy was identified as a significant favourable factor for both OS and PFS in both the whole and matched cohort.

**Conclusions:**

Induction chemotherapy followed by IRE is superior to induction chemotherapy followed by radiotherapy for treating LAPC. A randomized clinical trial comparing the efficacy of IRE and radiotherapy after the induction chemotherapy is therefore considerable.

## Background

Pancreatic cancer is the fourth leading cause of cancer-related death and is anticipated to emerge as the second leading cause of cancer-related death by 2030 [1]. Surgery offers the only chance of cure while only 10% of patients are candidates for surgical resection. Approximately 50% of patients present with metastatic disease and the remaining patients (40%) present vascular involvement prohibiting upfront resection, known as locally advanced pancreatic cancer (LAPC) [2–4]. The late diagnosis and aggressive nature of cancers contribute to the poor survival of patients with pancreatic cancer, which has changed little over the past two decades [5]. Moreover, the prognosis of patients with LAPC has remained poor due to high rates of local and distant tumor progression. The median survival of these patients is less than 1 year and five-year overall survival (OS) rate is less than 5% [6]. The current standard care for LAPC comprises a multidisciplinary approach, which includes chemotherapy alone, chemoradiotherapy and radiotherapy after induction chemotherapy, followed by reassessment for resectability [3, 7]. Although the superiority of chemotherapy, especially FOLFIRINOX-based regimen (5-fluorouracil, leucovorin, irinotecan, and oxaliplatin), demonstrated in the metastatic or resectable pancreatic cancer had led to many explorations of modified FOLFIRINOX-based chemotherapy in LAPC [8, 9], the induction chemotherapy combined with radiotherapy or not for LAPC seldom result in sufficient downstaging to enable potential curative resection to be attempted. Moreover, the role of radiotherapy was shown to be controversial and several randomized trials comparing chemotherapy and chemoradiotherapy in patients with LAPC demonstrated divergent conclusions [10–12]. Compared with chemotherapy alone, higher rates of toxicity and increased cost of chemoradiotherapy also contributed to dispute of the role of radiotherapy in the treatment of LAPC [12]. This unmet need prompted many researchers to examine novel treatments and optimize the current therapeutic approaches.

Although distant metastasis is the main form of disease progression of LAPC, many reports showed that locally destructive disease, rather than distant metastasis, contributed to 30–40% of deaths in patients with LAPC [13], which indicated the importance of local destructive therapies. Irreversible electroporation (IRE) is a novel local destructive therapy and is based on the transmission of short and high-voltage direct current pulses through the tumor, leading to changes of irreversible permeabilization in cell membrane integrity and subsequent apoptosis [14, 15]. Unlike radiofrequency ablation and microwave ablation, which will cause thermal damage to the nearby structures, such as blood vessels or bile ducts when they are applied in pancreas, IRE is a non-thermal ablative technique and it is not susceptible to the heat sink effect and then can be applied in areas around major blood vessels and vital structures [16]. Therefore, IRE is regarded as a safe and attractive treatment option for LAPC due to its unique feature of preserving important structures. Moreover, as a novel local destructive therapy, IRE was reported to benefit patients in terms of long-term survival when it was applied after the induction chemotherapy [17, 18]. However, nowadays, there is no evidence of the comparison of survival benefit between IRE and radiotherapy after induction chemotherapy in patients with LAPC. Therefore, we aimed to investigate the effect of these two local therapies after the induction chemotherapy on long-term OS and progression-free survival (PFS) and determine the prognostic predictors affecting survival outcomes in patients with LAPC.

## Methods

### Patients

All primary LAPC patients who were initially treated with IRE or radiotherapy after induction chemotherapy from August 2015 to August 2017 at Sun Yat-sen University Cancer Center were identified. A total of 96 patients were included using the following inclusion criteria: (1) pathologically confirmed pancreatic adenocarcinoma and radiologically confirmed LAPC. LAPC was defined as per the 7th edition of AJCC staging system for pancreatic cancer-described as arterial encasement of either the celiac axis or superior mesenteric artery, unreconstructable superior mesenteric or portal vein involvement, with no evident of metastatic disease from abdominal and thoracic computer tomograph (CT) [19]; (2) 4 months of induction chemotherapy (FOLFIRINOX or Gem-based chemotherapy) and no metastatic diseases. Twenty patients were excluded based on the following exclusion criteria: (1) other initial treatments, including surgical resection and radiofrequency ablation before or after chemotherapy (eight patients); (2) existing metastatic implants (nine patients); (3) a history of heart arrhythmia (one patient); (4) a history of second primary malignant tumors (two patients). Finally, 76 patients were included into this study.

### Data collection

All clinical and radiological data for diagnosis were retrieved from medical record archived at Sun Yat-sen University Cancer Center. The following data were collected and analyzed: age, gender, tumor size, tumor grade, lymph node (LN) metastasis, tumor site, tumor-node-metastasis (TNM) stage, white blood cell (WBC) count, hemoglobin (HGB), platelet (PLT) count, serum levels of alanine transaminase (ALT), aspartate aminotransferase (AST), alkaline phosphatase (ALP), glutamyl transpeptidase (GGT), albumin (ALB), total bilirubin (TBIL), indirect bilirubin (IBIL), C-reactive protein (CRP), carcinoembryonic antigen (CEA), carbohydrate antigen 19–9 (CA19–9), chemotherapy, radiotherapy and IRE treatment.

### Treatment procedure

The induction chemotherapy of either a FOLFIRINOX-based or GEM-based chemotherapy was used for 4 months in duration (total of three cycles of GEM-based chemotherapy or four to six cycles of FOLFIRINOX-based chemotherapy). After induction chemotherapy, CT, or magnetic resonance imaging was obtained with positron emission tomography, CA19–9 and CEA measurement to detect whether metastatic disease had occurred or not. IRE or radiotherapy was performed in patients when metastatic diseases were not detected. The same line of chemotherapy was performed after IRE or radiotherapy as the standard treatment if no complications or contraindication were reported. Radiotherapy delivered a median dose of 67 Gy in 30 daily fraction over 6 weeks. A planning CT was performed for every patient and the target volume delineation and organ at risk constrains followed the guidelines from National Comprehensive Cancer Network [3] and European Society for Medical Oncology [5].

The NanoKnife IRE equipment from Angiodynamics System (Queensbury, NY, USA) was used. IRE was performed in an open technique and general anesthesia with deep neuromuscular block is adopted. Each probe was a 19-gauge needle with depth marking and an echogenic tip and adjustable active exposure length. During the procedure of IRE, a maximal exposure of 1.5 cm was adopted for the pancreas. Three to six probes would be used according to the size and location of the tumor to create an electric field around the tumor, which would finally cause nanoscale pore formation in the plasma membrane. The probes were placed through the transverse mesocolon in a caudal-to-cranial direction. Ultrasound was used to guide the placement of all probes and then adequate space between probes is confirmed. The generator unit software was used to analysis the probe configuration data of ultrasound and provided optimal voltage and pulse length delivery. A setting of 1500 V/cm was often used as initial setting, with a planned delivery of 90 pulses at a pulse length of 70 to 90 ms. When the electric pulse started, the ablation was monitored with ultrasound and the current intensity was assessed. The voltage and pulse length would be changed according to the above information to achieve the desired initial current of 15 to 30 mA. The stepwise increasing resistance would lead to a drop in insistence, which was considered to be an indicator of an adequate ablation. If a tumor size was larger than 1.5 cm in the axial plane, a pull-back technique with the same procedure was performed to cover the entire area of ablation [20].

### Follow-up

The first follow-up visit was performed approximately 1 month after IRE or radiotherapy to assess technique efficacy, and then patients were followed up every 2–3 months during the first year and every 3–6 months thereafter. Physical examination, serum CA19–9 and CEA analysis and at least one imaging examination (abdominal CT or magnetic resonance imaging) were performed for each follow-up. OS was defined as the duration from the date of treatment until death or the last follow-up. PFS was defined as the duration from the date of treatment until the date when tumor progression was diagnosed or last follow-up. The last follow-up was completed on September 30, 2018.

### Propensity score matching (PSM) analysis

PSM analysis was used to minimize selection bias and balance variables. Propensity score for all patients were estimated by a logistic regression model using the following characteristics as covariates: age, gender, tumor size, tumor grade, LN metastasis, CA19–9, CEA. A one-to-one nearest-neighbour matching algorithm with a caliper of 0.2 and without replacement was used [21]. PSM analysis was performed using the “MatchIt” package in R software.

### Statistical analysis

Continuous variables were compared using independent sample t-test and Mann-Whitney U-test. Binary categorical variables were compared using the chi-square test. OS and PFS curves were analyzed using the Kaplan-Meier method, and differences between groups were compared by the log-rank test. Multivariate analysis was performed using the Cox regression model for variables which were found to be significant in univariate analysis, and the prognostic factors of OS and PFS were determined. The associated corresponding 95% confidence intervals (CIs) were calculated. Two-tailed *P* values less than 0.05 were considered statistically significant. Survival curves were depicted using MedCalc software version 11.4.2.0 (MedCalc, Ostend, Belgium) and all statistical analyses were performed using the R statistical package (R software version 3.4.2; R Foundation for Statistical Computing, Vienna, Austria).

## Results

### Patient characteristics

A total of 76 LAPC patients were treated with IRE or radiotherapy after induction chemotherapy. Among them, after induction chemotherapy for 4 months, 36 patients received IRE treatment and 40 patients received radiotherapy. The thresholds of the clinical and radiological variables were used as the cutoff values for these variables. The baseline characteristics of these clinical and pathological variables before and after PSM analysis were listed in Table 1. Thirty-six pairs of patients were matched and compared in this analysis. The median age for patients in the IRE group and radiotherapy group were 59.5 years (range 45.0–87.0 years) and 60.0 years (range 36.0–79.0 years), respectively. Female patients were more than male patients in IRE group while male patients occupied a little more proportion in the radiotherapy group. Poorly differentiated tumor and LN metastasis were more common in IRE group while radiotherapy group appeared to be associated with heavier tumor burden such as more cases with larger tumors. There were 21 (58.3%) patients received FOLFIRINOX-based chemotherapy and 15 (41.7%) patients received Gem-based chemotherapy, which was similar to the patients in radiotherapy group. All clinical and radiological variables were balanced between two groups after PSM analysis.

**Table 1:** Comparisons of clinical and imaging characteristics of patients

Characteristic		Before PSM				After PSM			
		Chemotherapy + IRE	Chemotherapy + radiotherapy	Total number	*P* value	Chemotherapy + IRE	Chemotherapy + radiotherapy	Total number	*P* value
Total number		36	40	76		36	36	72	
Age (years)	≤60	20	24	44	0.817	20	20	40	1.000
	> 60	16	16	32		16	16	32	
Gender	Female	19	15	34	0.248	19	15	34	0.479
	Male	17	25	42		17	21	38	
Tumor size (cm)	≤2	1	2	3	0.623	1	2	3	0.790
	2~4	20	18	38		20	18	38	
	>4	15	20	35		15	16	31	
Tumor grade	Well	3	5	8	0.763	3	5	8	0.633
	Moderate	20	23	43		20	21	41	
	Poor	13	12	25		13	10	23	
LN metastasis	Absent	9	14	23	0.454	9	14	23	0.312
	Present	27	26	53		27	22	49	
Tumor site	Head	18	18	36	0.498	18	16	34	0.562
	Body	15	15	30		15	14	29	
	Tail	3	7	10		3	6	9	
TNM stage	IIB	4	9	13	0.232	4	8	12	0.343
	III	32	31	63		32	28	60	
WBC (*10^9^)	≤10	32	37	69	0.702	32	33	65	0.989
	> 10	4	3	7		4	3	7	
HGB (g/L)	≤120	1	7	8	0.059	1	6	7	0.107
	>120	35	33	68		35	30	65	
PLT (*10^9^)	≤300	31	34	65	0.978	31	31	62	1.000
	>300	5	6	11		5	5	10	
ALT (U/L)	≤40	26	31	57	0.608	26	28	54	0.786
	> 40	10	9	19		10	8	18	
AST (U/L)	≤40	29	34	63	0.762	29	31	60	0.753
	>40	7	6	13		7	5	12	
ALP (U/L)	≤100	19	28	47	0.158	19	25	44	0.227
	>100	17	12	29		17	11	28	
GGT (U/L)	≤45	19	22	41	0.935	19	20	39	1.000
	>45	17	18	35		17	16	33	
ALB (g/L)	≤40	4	4	8	0.957	4	4	8	1.000
	>40	32	36	68		32	32	64	
TBIL (umol/L)	≤20.5	27	33	60	0.574	27	30	57	0.563
	>20.5	9	7	16		9	6	15	
IBIL (umol/L)	≤15	32	39	71	0.184	32	35	67	0.357
	>15	4	1	5		4	1	5	
CRP (ng/L)	≤3	24	24	48	0.637	24	21	45	0.627
	>3	12	16	28		12	15	27	
CEA (ng/mL)	≤5	21	25	46	0.815	21	22	43	0.982
	>5	15	15	30		15	14	29	
CA19–9 (U/ml)	≤35	9	9	18	0.962	9	8	17	0.987
	>35	27	31	58		27	28	55	
Chemotherapy	FOLFIRINOX	21	18	39	0.262	21	17	38	0.479
	Gem	15	22	37		15	19	34	

*IRE* irreversible electroporation, *LN* lymph node metastasis, *TNM* tumor-node-metastasis stage, *WBC* white blood cell count, *PLT* platelet count, *ALT* alanine transaminase, *AST* aspartate aminotransferase, *ALP* alkaline phosphatase, *GGT* glutamyl transpeptidase, *ALB* albumin, *TBIL* total bilirubin, *IBIL* indirect bilirubin, *CRP* C-reactive protein, *CEA* carcinoembryonic antigen, *CA*19–9 carbohydrate antigen 19–9

### Survival and tumor progression in all patients

**Fig. 1:**
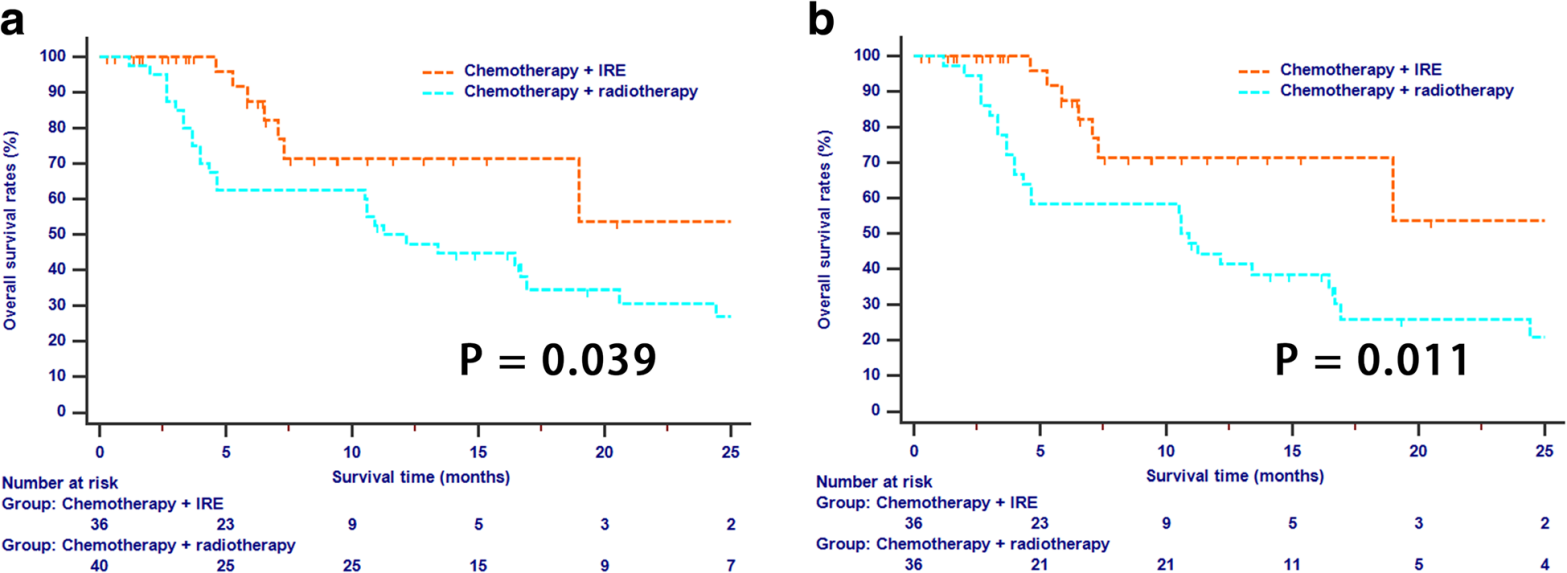
The Kaplan-Meier survival curves of overall survival stratified by treatment strategies for patients with LAPC before (**a**) and after (**b**) propensity score matching Abbreviations: LAPC, locally advanced pancreatic cancer.

The median follow-up time duration was 10.0 months (range 1.2–30.6 months) for the whole study cohort. During the follow-up period, 7 (19.4%) patients in the IRE group and 27 (75%) patients in the radiotherapy group had died (*p* < 0.001). Before PSM analysis, the median OS in the IRE and radiotherapy group were 21.6 and 11.3 months, respectively while the 1- and 2-year OS rates in these two groups were 71.4, 53.5 and 47.2%, 26.9%, respectively (*p* = 0.039, Fig. 1a). In addition, patients in IRE group also had significant longer OS than that of patients in radiotherapy group after PSM analysis (median OS, 21.6 months vs. 10.6 months; 1-year OS rate, 71.4% vs. 41.3%; 2-year OS rate, 53.5% vs. 20.7%; *p* = 0.011, Fig. 1b).

**Fig. 2:**
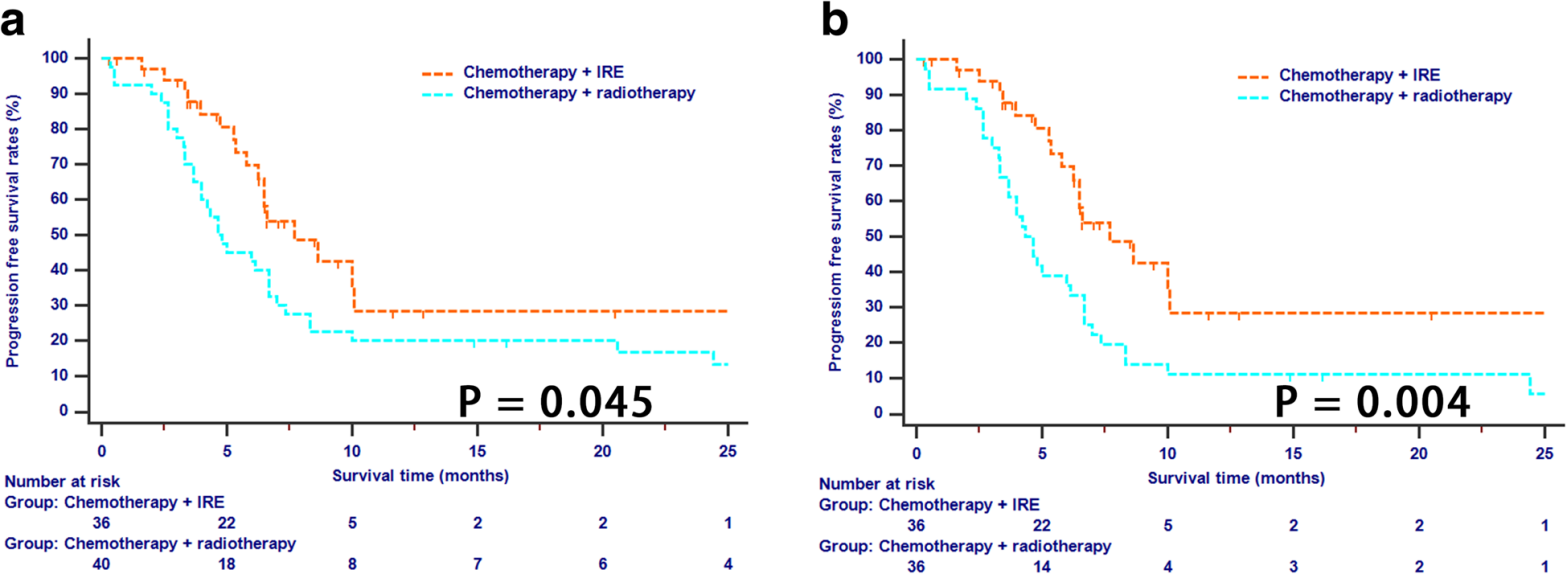
The Kaplan-Meier survival curves of progression free survival stratified by treatment strategies for patients with LAPC before (**a**) and after (**b**) propensity score matching. Abbreviations: LAPC, locally advanced pancreatic cancer

There were a total of 50 patients who had tumor progression during the study cohort, including 17 (47.2%) patients in IRE group and 33 (91.6%) patients in radiotherapy group (*p* < 0.001). The median PFS for patients in IRE and radiotherapy group were 7.7 months and 4.7 months, respectively (*p* = 0.045, Fig. 2a). The same results were also obtained in patients of two groups after PSM (median PFS, 7.7 months vs. 4.3 months; 1-year PFS rate, 28.4% vs. 11.1%; 2-year PFS rate, 28.4% vs. 5.6%; *p* = 0.004, Fig. 2b). It was indicated that although patients in two groups had tumors with similar clinical and radiological features, tumors were more inclined to progress in patients in radiotherapy group, compared with those in IRE group.

### Prognostic factors associated with OS and PFS

All clinical and radiological variables were included in Cox regression analysis. Univariate analysis for OS revealed that IRE treatment (IRE vs radiotherapy, hazard ratio (HR) = 0.355; 95% CI, 0.154–0.822; *p* = 0.016), CRP (> 3 ng/L vs ≤ 3 ng/L; HR = 3.939, 95% CI, 1.867–8.311, *p* < 0.001) and ALB (> 40 g/L vs ≤ 40 g/L; HR = 0.35, 95% CI, 0.128–0.934, *p* = 0.036) were associated with OS. Moreover, independent prognostic factor identified by multivariate analysis included chemotherapy followed by IRE treatment (HR = 0.362; 95% CI, 0.153–0.854; *p* = 0.020) and lower CRP level (HR = 3.432, 95% CI, 1.628–7.238, *p* = 0.001) (Table 2). Univariate and multivariate analyses were also applied for PFS analysis. It was shown that IRE treatment (IRE vs radiotherapy, HR = 0.435; 95% CI, 0.242–0.783; *p* = 0.005), gender (male vs female; HR = 2.284, 95% CI, 1.269–4.112, *p* = 0.006) and CA19–9 (> 35 U/mL vs ≤ 35 U/mL; HR = 2.530, 95% CI, 1.179–5.429, *p* = 0.017) were associated with PFS. In addition, only chemotherapy followed by IRE treatment (HR = 0.438; 95% CI, 0.243–0.790; *p* = 0.006) was identified to be an independent favourable factor for PFS in patients with LAPC (Table 3).

**Table 2:** Univariate and multivariate analyses of OS in patients

Characteristic		Before PSM						After PSM					
		Univariate analysis			Multivariate analysis			Univariate analysis		Multivariate analysis			
		HR	95%CI	P	HR	95%CI	P	HR	95% CI	P	HR	95% CI	P
Age (years)	≤60/> 60	1.718	0.880–3.356	0.113			NI	1.443	0.734–2.837	0.288			NI
Gender	Female / Male	1.537	0.764–3.093	0.228			NI	1.930	0.953–3.908	0.068			NI
Tumor size (cm)	≤2/2~4/>4	1.158	0.636–2.108	0.631			NI	1.404	0.763–2.584	0.275			NI
Tumor grade	Well / Moderate / Poor	1.030	0.597–1.778	0.915			NI	1.188	0.681–2.072	0.545			NI
LN metastasis	Absent / Present	0.677	0.332–1.379	0.282			NI	0.787	0.386–1.604	0.509			NI
Tumor site	Head / Body / Tail	0.942	0.575–1.544	0.814			NI	1.003	0.598–1.681	0.992			NI
WBC (*10^9^)	≤10/> 10	1.008	0.354–2.872	0.988			NI	0.878	0.305–2.525	0.809			NI
HGB (g/L)	≤120/> 120	0.656	0.254–1.693	0.383			NI	0.445	0.169–1.174	0.102			NI
PLT (*10^9^)	≤300/> 300	1.220	0.503–2.957	0.660			NI	1.521	0.614–3.764	0.365			NI
ALT (U/L)	≤40/>40	0.768	0.348–1.696	0.514			NI	0.790	0.356–1.752	0.562			NI
AST (U/L)	≤40/>40	0.497	0.175–1.410	0.189			NI	0.540	0.190–1.537	0.248			NI
ALP (U/L)	≤100/>100	0.919	0.456–1.854	0.813			NI	0.905	0.446–1.840	0.784			NI
GGT (U/L)	≤45/>45	0.898	0.459–1.760	0.755			NI	0.989	0.501–1.950	0.974			NI
ALB (g/L)	≤40/> 40	0.317	0.117–0.857	0.024	0.363	0.130–1.012	0.053	0.346	0.128–0.934	0.036	0.389	0.141–1.076	0.069
TBIL (umol/L)	≤20.5/>20.5	0.649	0.251–1.675	0.371			NI	0.715	0.275–1.855	0.490			NI
IBIL (umol/L)	≤15/>15	0.483	0.066–3.557	0.475			NI	0.437	0.059–3.215	0.416			NI
CRP (ng/L)	≤3/>3	4.040	1.974–8.266	<0.001	3.567	1.741–7.309	0.001	3.939	1.867–8.311	<0.001	3.432	1.628–7.238	0.001
CEA (ng/mL)	≤5/>5	1.372	0.687–2.741	0.370			NI	1.414	0.702–2.850	0.333			NI
CA19–9 (U/ml)	≤35/>35	1.650	0.720–3.782	0.237			NI	2.056	0.848–4.987	0.111			NI
Chemotherapy	With IRE/ With radiotherapy	0.424	0.183–0.892	0.045	0.422	0.177–0.998	0.049	0.355	0.154–0.822	0.016	0.362	0.153–0.854	0.020
Cheotherapy type	FOLFIRINOX/Gem	0.834	0.596–1.168	0.291			NI	0.864	0.613–1.219	0.407			NI

Abbreviations as in Table 1

**Table 3:** Univariate and multivariate analyses of PFS in patients

Characteristic		Before PSM						After PSM					
		Univariate analysis			Multivariate analysis			Univariate analysis		Multivariate analysis			
		HR	95%CI	P	HR	95%CI	P	HR	95%CI	P	HR	95% CI	P
Age (years)	≤60/> 60	1.178	0.674–2.058	0.566			NI	0.956	0.547–1.671	0.874			NI
Gender	Female / Male	1.587	0.905–2.783	0.107			NI	2.284	1.269–4.112	0.006	1.798	0.966–3.330	0.064
Tumor size (cm)	≤2/2~4/>4	0.787	0.490–1.265	0.324			NI	0.970	0.596–1.579	0.903			NI
Tumor grade	Well / Moderate / Poor	1.135	0.735–1.753	0.568			NI	1.333	0.855–2.079	0.205			NI
LN metastasis	Absent / Present	0.900	0.501–1.614	0.723			NI	1.078	0.600–1.938	0.801			NI
Tumor site	Head / Body / Tail	0.935	0.621–1.407	0.748			NI	0.995	0.650–1.525	0.983			NI
WBC (*10^9^)	≤10/>10	1.149	0.486–2.718	0.751			NI	1.019	0.431–2.411	0.965			NI
HGB (g/L)	≤120/>120	0.685	0.308–1.527	0.355			NI	0.477	0.211–1.076	0.075			NI
PLT (*10^9^)	≤300/>300	0.613	0.261–1.438	0.261			NI	0.676	0.287–1.592	0.370			NI
ALT (U/L)	≤40/>40	0.729	0.373–1.426	0.356			NI	0.737	0.375–1.447	0.375			NI
AST (U/L)	≤40/>40	0.662	0.309–1.417	0.288			NI	0.691	0.320–1.490	0.345			NI
ALP (U/L)	≤100/>100	0.823	0.459–1.476	0.514			NI	0.790	0.439–1.423	0.433			NI
GGT (U/L)	≤45/>45	0.693	0.390–1.230	0.210			NI	0.741	0.414–1.327	0.313			NI
ALB (g/L)	≤40/>40	0.602	0.253–1.429	0.250			NI	0.666	0.281–1.582	0.357			NI
TBIL (umol/L)	≤20.5/>20.5	0.691	0.336–1.423	0.316			NI	0.721	0.350–1.487	0.376			NI
IBIL (umol/L)	≤15/>15	0.884	0.317–2.461	0.813			NI	0.769	0.276–2.144	0.616			NI
CRP (ng/L)	≤3/>3	1.840	1.030–3.286	0.040	1.586	0.877–2.866	0.127	1.763	0.970–3.202	0.063			NI
CEA (ng/mL)	≤5/>5	1.466	0.835–2.572	0.183			NI	1.464	0.829–2.586	0.189			NI
CA19–9 (U/ml)	≤35/>35	2.078	1.009–4.279	0.047	1.908	0.917–3.968	0.084	2.530	1.179–5.429	0.017	2.032	0.904–4.569	0.086
Chemotherapy	With IRE/ With radiotherapy	0.557	0.310–1.000	0.050	0.582	0.322–0.950	0.048	0.435	0.242–0.783	0.005	0.438	0.243–0.790	0.006
Cheotherapy type	FOLFIRINOX/Gem	0.965	0.733–1.271	0.803			NI	1.014	0.768–1.340	0.921			NI

Abbreviations as in Table 1

### Comparisons of toxicities and complications after treatment

Patients of two groups were evaluated for toxicity. The most frequently reported toxicities were hypoalbuminemia and hypotension for patients in IRE group while hematologic adverse events, such as neutropenia, lymphopenia and fatigue, vomiting, and diarrhea were significantly more frequently observed in radiation group. Occurrences of complications were less in patients in IRE group, even though the significances were not significant due to the limited cases. However, muscle weakness occurred significantly more often in the radiation group (10 of 36 patients) (*p* = 0.006) (Table 4).

**Table 4:** Comparison of toxicity and complications between two groups

Complications	Chemotherapy + IRE	Chemotherapy + radiotherapy	*P* value
All	36	36	
Toxicity			
Neutropenia	1	12	0.001
Lymphopenia	2	10	0.024
Hypoalbuminemia	3	7	0.307
Hypotension	3	5	0.710
Hypokalemia	2	7	0.151
Fatigue	2	10	0.024
Vomiting	0	9	0.002
Diarrhea	2	9	0.046
Complications			
Thrombosis	4	5	0.890
Ascites	1	6	0.107
Abdominal pain	2	8	0.085
Muscle weakness	1	10	0.006

Abbreviations as in Table 1

## Discussion

LAPC, which represents nearly 40% of all pancreatic cancers, is a devastating disease with relatively high mortality rates and five-year survival rate lower than 5% [6, 22]. Recently, the biological aggressiveness and the nature history of LAPC have been questioned and then LAPC are believed to have a different prognosis compared with metastatic pancreatic cancer. The progress of chemotherapy in the treatment of metastatic and resectable pancreatic cancer provided new insight into the treatment of LAPC. A recent meta-analysis of retrospective studies showed that FOLFIRINOX-based chemotherapy could prolong survival for patients with PDAC [23]. Another interim result from a phase II trial also illustrated that 21% of patients with LAPC were deemed eligible for surgery after 6 cycles of Gemcitabine-based chemotherapy [24]. As many clinical trials were conducted in patients with advanced diseases, which included many cases of distant metastatic diseases, there were no randomized-controlled trial data available, specially demonstrating the benefit of chemotherapy in patents with LAPC. However, recent studies had also consolidated the foundation role of chemotherapy in the treatment of LAPC for its potential to develop metastasis. Moreover, apart from distant metastasis, a good proportion of patients may die from local tumor progression. It was reported that fully 28% of patients with LAPC had no evidence of metastasis at the time of death in a recent rapid autopsy from Johns Hopkins [25]. Local tumor progression was a main cause which was responsible for death of patient with LAPC. On the basis of systemic therapies which can improve the control of microscopic disease, strategies to optimize local control may play an increasing role in maximizing therapy [26, 27].

As a local treatment, radiotherapy plays a role in the localized control of LAPC. Chemotherapy followed by chemoradiotherapy is an option in patients with LAPC, demonstrating stability than chemotherapy alone. This approach aims to improve local control and may achieve downstaging of tumors [16]. However, current clinical trials comparing chemotherapy with chemoraditherapy have reported mixed responses. A large retrospective study from MD Anderson showed that stable diseases following induction chemotherapy demonstrated survival benefit from chemoradiotherapy [28] while it was shown that patient with LAPC experienced significantly shorter OS and more common toxicity after chemotherapy followed by radiotherapy, compared with single chemotherapy in the FFCDSFRO study [10]. Therefore, there was no concensus concerning the survival benefit of radiotherapy in patients with LAPC based on the current evidence and more clinical trials were needed in light of the fact that most patients still progressed with metastatic disease developing after chemoradiotherapy.

IRE is a novel local control treatment which has been recently applied in the treatment of LAPC. The safety and effectiveness of IRE have been reported by many previous studies [14, 29]. Moreover, it was illustrated that IRE was reasonably safe in LAPC after chemotherapy and with promising results regarding efficacy [17, 30]. Although it was shown that the subsequent IRE or radiotherapy after chemotherapy might result in improved survival compared with chemotherapy alone, there was no direct data illustrating the comparison of efficacy of these two local control treatments after the induction chemotherapy in patients with LAPC. In this study, it was shown that the median OS was 21.6 months in patients after the induction chemotherapy and IRE, which was similar with the expected survival in study conducted by Huang et al. [30]. It was significantly higher than that of patients in the radiotherapy group, showing a promising improvement for patients with LAPC. PSM analysis, which could reduce the confounding bias of baseline characteristics, was applied in this study. The median OS for patients receiving chemotherapy following by IRE and radiotherapy were 21.6 and 11.3 months, respectively. The survival benefit was even more obvious between these groups after PSM analysis. Moreover, across-study comparisons were also conducted in this study. For example, Krishan et al. showed a median OS of 11.9 months after chemotherapy followed by chemoradiotherapy [28]. Another similar study also indicated that median OS was 11.7 months in patients receiving induction chemotherapy followed by radiotherapy [31]. The comparison of the survival further consolidated the survival advantage of IRE therapy compared with radiotherapy after the induction chemotherapy.

Compared with progressed disease observed in 33 out of 36 patients in radiotherapy group, only half of patients in IRE group had disease progression during the follow-up period. In addition, significant higher PFS was observed in patients after induction therapy followed by IRE, compared with radiotherapy. Multivariate analysis further illustrated IRE was an independent favourable factor for long-term OS and RFS, showing the survival benefit from IRE for patients with LAPC. As a local destructive treatment, IRE was helpful of chemotherapy delivery to tumor by disrupting the dense stroma of pancreatic cancer [32, 33]. In addition, due to the feature of non-thermal ablation, electric field of extremely high voltage can be applied through the whole tumor without harming nearby important structure in IRE treatment. In contrast, the duodenum and small intestine were easily harmed by high doses of radiation. Conventional radiotherapy was used most frequently in the treatment of LAPC and one of the reasons that studies failed to demonstrate the superiority of radiotherapy was the insufficient dose of radiation [34]. Therefore, compared with radiotherapy, IRE is more active in inducing tumor destruction and providing survival benefit for patients with LAPC. Intensity modulated radiation therapy (IMRT) and stereotactic body radiation therapy (SBRT) are two new delivery systems. They have theoretical advantages over conventional external beam radiation that both delivered more focused doses of radiation while spared normal tissue and leaved less toxicity [35, 36]. The increasing use of these novel radiation therapies may further improve survival of patients with LAPC. Interestingly, gender tended to be prognostic factor for survival. In this study, higher values of CRP were more frequently observed in male patients (16 of 36, 44.4%), compare with female patients (9 of 36, 25.0%). Thus, the multicollinearity might contribute to seeming prognostic significance of gender. Additionally, multivariate analysis revealed that gender failed to significantly predict survival in LAPC patients, which was inconsistent with previous studies [13, 37].

The most encouraging result of patients with LAPC after IRE treatment was reported by Martin et al. [37], in which with a median follow-up of 29 months, the median OS was 24.9 months and the median PFS was 12.4 months. Different from our study, in Martin’s study, 25% of patients had received resection and margin accentuation by IRE and conventional chemotherapy and radiotherapy were conducted in all patients. Compared with Martin’s study, although the reported median OS and PFS were similar, patients experienced significantly more progressed diseases in our study. This result illustrated that chemotherapy was needed to control microscopic disease and distant metastasis even after local control of disease by IRE. Maybe only a multidisciplinary approach can be effective in obtaining both a local tumor reduction and a systemic control of disease.

There were several limitations which should be considered. First, the main limitation of this study was that the sample size of patients was small to draw definitive conclusions. Second, potential patient selection bias could not be completely avoided even after PSM analysis. Third, most of the radiotherapy was conventional radiation therapy, IMRT or SBRT might induce a better local control of diseases as mentioned above. Further prospective studies are needed to confirm the results of this study.

## Conclusion

In conclusion, after induction chemotherapy, IRE resulted in better long-term OS and PFS than radiotherapy in patients with LAPC and the induction chemotherapy followed by IRE could be considered as a suitable treatment modality. A randomized clinical trial comparing the efficacy of IRE and radiotherapy after the induction chemotherapy, is therefore considerable.

## Abbreviations

ALB: albumin
ALP: alkaline phosphatase
ALT: alanine transaminase
AST: aspartate aminotransferase
CA19–9: carbohydrate antigen
CEA: carcinoembryonic antigen
CI: confidence interval
CRP: C-reactive protein
CT: computer tomograph
GGT: glutamyl transpeptidase
HGB: hemoglobin
HR: hazard ratio
IBIL: indirect bilirubin
IMRT: Intensity modulated radiation therapy
IRE: irreversible electroporation
LAPC: locally advanced pancreatic cancer
LN: lymph node
OS: overall survival
PFS: progression-free survival
PLT: platelet
PSM: Propensity score matching
SBRT: stereotactic body radiation therapy
TBIL: total bilirubin
TNM: tumor-node-metastasis
WBC: white blood cell
